# High-temperature operation of gallium oxide memristors up to 600 K

**DOI:** 10.1038/s41598-023-28075-4

**Published:** 2023-01-30

**Authors:** Kento Sato, Yusuke Hayashi, Naoki Masaoka, Tetsuya Tohei, Akira Sakai

**Affiliations:** Graduate School of Engineering Science, Osaka University, 1-3 Machikaneyama-Cho, Toyonaka, Osaka 560-8531 Japan

**Keywords:** Electrical and electronic engineering, Materials for devices, Electronic and spintronic devices

## Abstract

Memristors have attracted much attention for application in neuromorphic devices and brain-inspired computing hardware. Their performance at high temperatures is required to be sufficiently reliable in neuromorphic computing, potential application to power electronics, and the aerospace industry. This work focuses on reduced gallium oxide (GaO_*x*_) as a wide bandgap memristive material that is reported to exhibit highly reliable resistive switching operation. We prepared amorphous GaO_*x*_ films to fabricate Pt/GaO_*x*_/indium tin oxide memristors using pulsed laser deposition. Stable resistive switching phenomena were observed in current–voltage properties measured between 300 and 600 K. The conduction mechanism analysis revealed that the resistive switching is caused by the transition between ohmic and space charge limiting current conductions. We elucidated the importance of appropriate control of the density of oxygen vacancies to obtain a high on/off resistance ratio and distinct resistive switching at high temperatures. These results indicate that GaO_*x*_ is a promising memristor material that can be stably operated even at the record-high temperature of 600 K.

## Introduction

Metal-oxide memristors that exhibit resistive switching via oxide ion migration have attracted much attention for use in resistive random-access memories, neuromorphic devices, and brain-inspired computing hardware^1–4^. Metal-oxide memristors are driven by applying an external bias voltage that changes the distribution of oxygen vacancy ions in the thin film and determines the resistive state of the device^5–10^. One of the main principles of resistive switching in memristors is based on the filamentary conduction model by which the formation and rupture of conductive filaments in thin films change the resistance state^11–16^. Such filament-type memristors show sharp resistance changes and a high on/off ratio of resistance, whereas stochastic filament formation is likely to induce unintentional variabilities in performance. In contrast, a non-filamentary-type memristor has been reported to be driven by the homogeneous migration of ions, modifying the electronic carrier distribution in the memristor^17–28^. This allows for a deterministic bulk resistance change and is appropriate for realizing the highly reliable memristor operation required for mass production.

The high-temperature performance of memristors must be sufficiently reliable in neuromorphic computing and potential applications in power electronics and the aerospace industry. Static random-access memories consisting of several Si metal–oxide–semiconductor field-effect transistors are among the most competitive platforms for implementing artificial synaptic devices in neuromorphic semiconductor chips. However, the operating temperature of Si-based transistors is limited to less than 500 K, mainly because their bandgap energy (*E*_g_) is 1.1 eV^29^. This has motivated the exploration of wide bandgap materials for application in memristors that operate at high temperatures. Primary filament-type memristive materials, such as TiO_2_ (*E*_g_ = 3.0 eV) and HfO_2_ (*E*_g_ = 5.7 eV), have been studied by considering the temperature dependence of electrical properties, and thus far resistive switching operations have been reported at up to 373 K^30–34^. Recently, higher-temperature operation at 613 K has been demonstrated in filament-type MoS_2−*x*_O_*x*_ memristors fabricated on a two-dimensional material platform^35^. However, few reports on non-filamentary-type memristors have been published on either temperature-dependent electrical characteristics or high-temperature operation.

This study focuses on reduced gallium oxide (GaO_*x*_) with an amorphous phase as a wide bandgap, non-filament-type memristive material. Amorphous GaO_*x*_ has been reported to exhibit bandgap energy of 4.1 eV^36^ and non-filamentary-type conduction driven by the bias application^17–19,21,27,28^, indicating its significant potential for reliable high-temperature operation. Furthermore, the amorphous structure provides flexibility in the selection of substrates and compatibility with the monolithic fabrication process for integrated circuits, such as the previously demonstrated crossbar-array architecture^27,37^. The present study reports the resistive switching operation of GaO_*x*_ memristors at a record high temperature of 600 K. Analysis of the electrical conduction mechanism revealed that resistive switching obeys Ohm’s law in a low-resistance state (LRS) and the Mott–Gurney law in a high-resistance state (HRS). The essence of the high-temperature operation is to control moderately the density of oxygen vacancies in GaO_*x*_ for abrupt switching to occur between the LRS and HRS.

## Results and discussion

### Physical and chemical properties of GaO_***x***_

**Figure 1:**
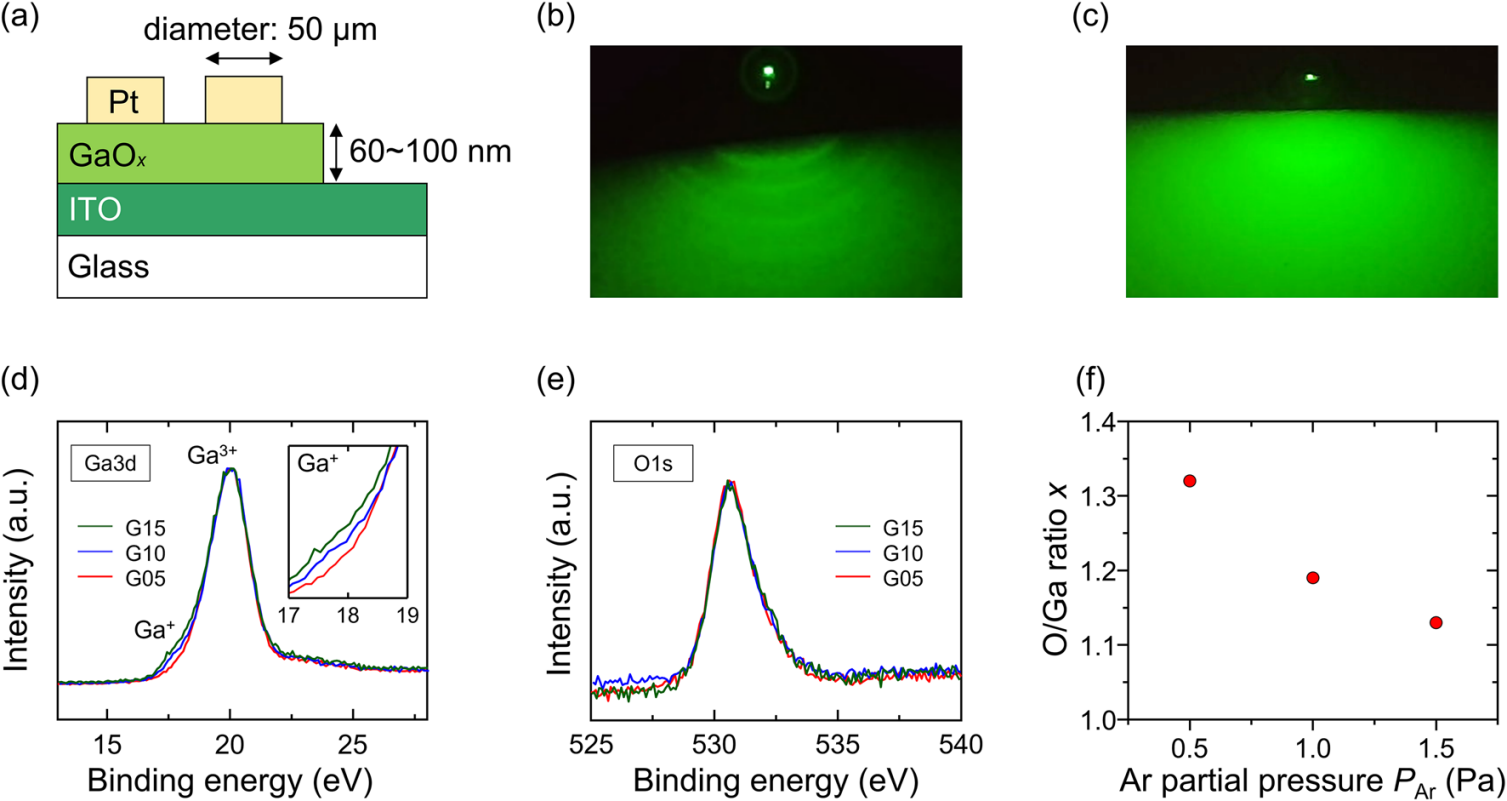
(**a**) Cross-sectional schematic of fabricated Pt/GaO_*x*_/ITO memristor. RHEED patterns of surfaces of (**b**) ITO and (**c**) post-deposition Sample G05. XPS spectra of (**d**) Ga3d and (**e**) O1s, and (**f**) the O/Ga ratio as a function of *P*_Ar_ during PLD.

A schematic of the fabricated device structure is shown in Fig. 1a. GaO_*x*_ films were deposited on indium tin oxide (ITO)-coated glass substrates by pulsed laser deposition (PLD) at room temperature. Three types of samples were prepared by changing the Ar partial pressure (*P*_Ar_) between 0.5, 1.0, and 1.5 Pa during deposition to control the O/Ga ratio (hereafter named Samples G05, G10, and G15, respectively). The reflection high-energy electron diffraction (RHEED) patterns were observed in situ to confirm the amorphous phase of GaO_*x*_.

Figure 1b,c show the RHEED patterns of the ITO/glass substrate and post-deposition Sample G05, respectively. The halo pattern of amorphous GaO_*x*_ covered the ring pattern of polycrystalline ITO. Samples G10 and G15 exhibited similar patterns of amorphous GaO_*x*_. The results of X-ray photoelectron spectroscopy (XPS) for the Ga3d and O1s peaks are shown in Fig. 1d,e, respectively. In general, the Ga3d peak was composed of major Ga^3+^ (20.7 eV) and minor Ga^+^ (19.6 eV) peaks^18^. In the present case, the latter is pronounced, particularly in sample G15, indicating that the sample is in the most highly reduced state. The O/Ga ratio, composition *x* in GaO_*x*_, was calculated from the ratio of the Ga and O peak intensities, and the results were plotted as a function of *P*_Ar_ during deposition, as shown in Fig. 1f. The resultant values of 1.3, 1.2, and 1.1 for Samples G05, G10, and G15, respectively, were close to the value of *x* = 1.1–1.3 found in previous studies^17,18,21^ where the non-filamentary-type memristor was reported. These results indicate that during PLD, Ar atoms are the primary scattering source of light O atoms that are laser-ablated from the target and reach the substrate, whereas the heavy Ga atoms are less scattered. Thus, the higher the *P*_Ar_ is, the more highly reduced the GaO_*x*_, leading to an increase in the density of the oxygen vacancies in the film.

### Electrical property at room temperature

**Figure 2:**
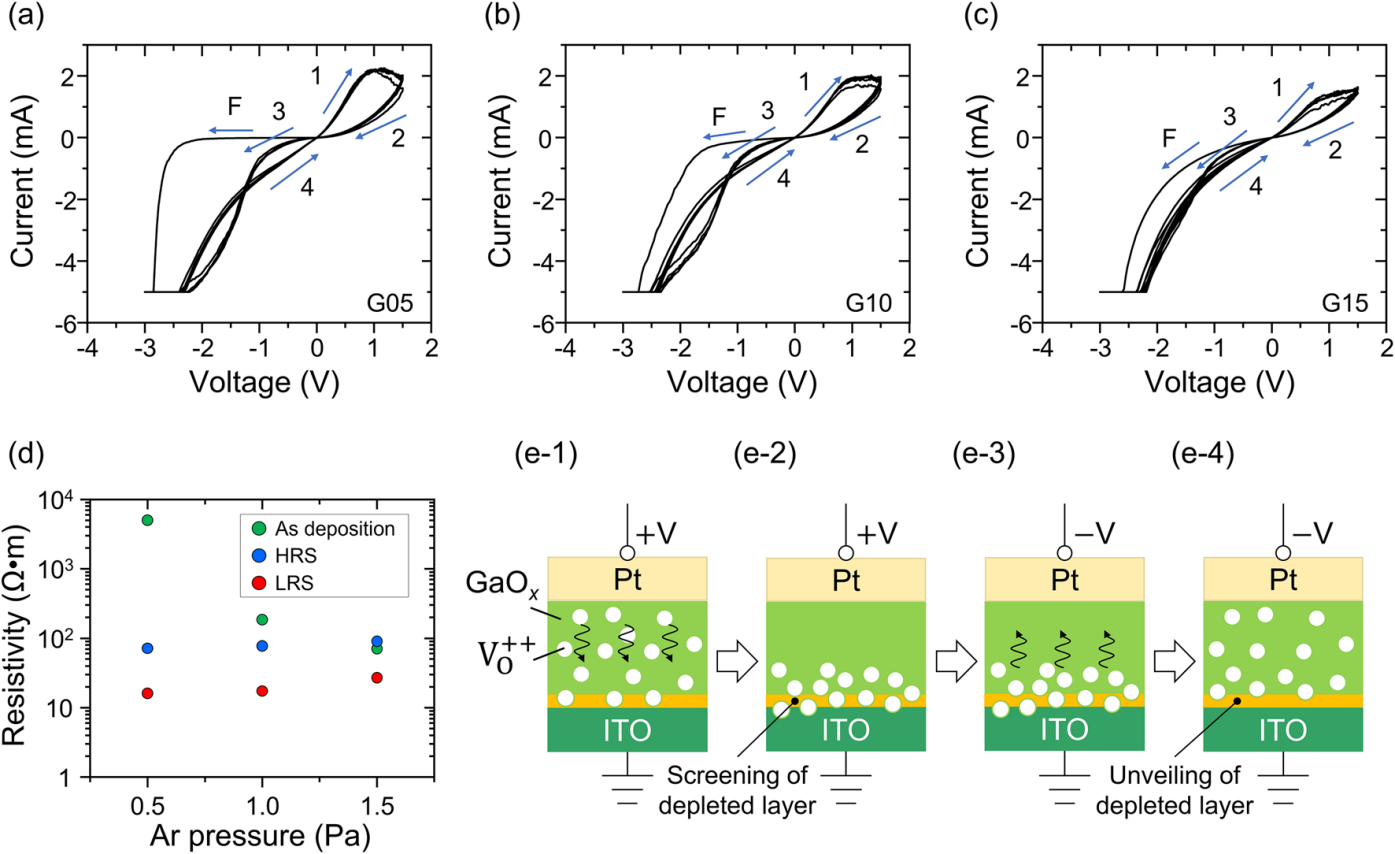
*I–V* curves obtained at room temperature for Samples (**a**) G05, (**b**) G10, and (**c**) G15. (**d**) Measured resistivity of the GaO_*x*_ films before switching the resistance at the HRS and the LRS depending on *P*_Ar_ during PLD. (**e**) Schematics of oxygen vacancy distribution in the memristor corresponding to the situation of the voltage sweep through Regions 1–4.

The resistive switching behavior of the samples was first characterized at room temperature. A single voltage sweep cycle was performed with a sweep rate of 1.1 V/s and current compliance of − 5 mA for 100 cycles. Figure 2a–c show the current–voltage (*I–V*) curves of the first 10 cycles for Samples G05, G10, and G15, respectively. See Supplementary Fig. S1 of Supplementary Information for all 100 cycles of the *I*–*V* curve. The first *I–V* curve at a negative voltage sweep (0 →  − 3 → 0 V) is marked by arrow F, and the subsequent sweep direction of the hysteresis *I–V* loop (0 →  + 1.5 → 0 →  − 3 → 0 V) is depicted by arrows 1–4 in the figures; hereafter, these curves are referred to as Regions F and 1–4, respectively. Every *I–V* curve shows the counter figure-8 (CF8) feature in the first loop: the device is SET to a LRS by applying a negative voltage and then RESET to a HRS by applying a positive voltage.

As shown in Region F, a high turn-on voltage was required for the first SET operation. This is probably because an electrical barrier is present at the Pt/GaO_*x*_ interface, which is formed by the slight oxidation of the GaO_*x*_ surfaces just before Pt sputtering during sample fabrication. It is noteworthy that an *I–V* sweep starting from the positive voltage (0 →  + 1.5 → 0 V) showed little current flow and no hysteresis characteristics at 1.5 V. The subsequent negative voltage sweep (0 →  − 3 → 0 V) reproduced the hysteresis *I*–*V* curve with region F. For the details, see Supplementary Fig. S2 of Supplementary Information for the *I–V* characteristics starting from the negative and positive voltage sweeps. Since the change in the *I–V* curve from the first to the second loop is moderate, and the features of the hysteresis loops are similar to those of previous studies^17,18^, the resistive switching mechanism in the present devices is considered to be non-filamentary-type conduction. The effect of the electrical barrier is no longer observed after the first voltage cycle. Consequently, the turn-on voltage in the first SET operation decreased for samples with GaO_*x*_ films grown with increased *P*_Ar_.

Interfacial resistive switching is a non-filamentary conduction mechanism typically observed in perovskite, e.g., Pr_0.7_Ca_0.3_MnO_3_^38^. Even GaO_*x*_ memristors exhibited non-filamentary interfacial resistive switching; Guo et al. reported that the aggregation and diffusion of oxygen vacancies at the Schottky contact interface of Pt/GaO_1.3_ change the amount of tunnel current, leading to the forming-free resistance switching^21^. The present study could exhibit interfacial resistive switching at the Pt/GaO_*x*_ Schottky contact. As described above, the as-deposited GaO_*x*_ surface is prone to be slightly oxidized due to air exposure, forming relatively high resistive layers at the Pt/GaO_*x*_ interface. Therefore, a high turn-on voltage was required to initiate resistive switching. The reduction of turn-on voltage at Region F was distinct in Samples G10 and G15, indicating that abundant oxygen vacancy mitigated the carrier depletion at the as-deposited Pt/GaO_*x*_ interface. Through this first negative voltage application, we infer that the Pt/GaO_*x*_ interface was reduced via the supply of oxygen vacancies from the ITO side. Notably, the high turn-on voltage could form a non-uniform current path. Such high turn-on voltage is considered to induce the area-dependent leakage current, e.g., at the edge of the circular electrode, resulting in the in-plane inhomogeneous segregation of oxygen vacancy via resistive switching. Given this assumption, spatially non-uniform distribution of Pt/GaO_*x*_ Schottky barrier height potentially weakens the area dependency of current flow. This assumption is consistent with the no electrode-area dependency of output current observed in this study (see Supplementary Fig. S3 of Supplementary Information for electrode area dependency).

To support the non-filamentary conduction mechanism in the present study, we refer to the recent work of our group^27^; a scaling down of output current *I* against electrode area *S* was demonstrated via the fabrication of crossbar array amorphous GaO_*x*_ memristors, where the slopes of double logarithmic plot in *I*–*S* characteristic are 0.63 for LRS and 0.65 for HRS. We consider that the improved scaling down feature is attributable to the voltage application protocol, where a gradual increase in sweep range could help to avoid the high turn-on voltage and enhance the in-plane homogeneous current flow. A comprehensive investigation remains for future work (see Supplementary Fig. S4 of Supplementary Information for *I*–*S* characteristics).

Filamentary resistive switching in GaO_*x*_ was reported by Guo et al. using Pt/GaO_*x*_/Pt^39^ and by Shen et al. using Pt/GaO_*x*_/SiC/Pt^40^. Abrupt current jumps with a resistance ratio of more than 10^3^ were observed in both *I–V* characteristics, which is the distinct feature of filamentary resistive switching. In our device, neither such abrupt current jumps nor prominent resistance ratios were observed during the resistive switching; hence, it is likely that our device obeys a non-filamentary conduction mechanism. It is still hard to prove the absence of potentially formed conductive filaments from the Pt electrode side since the conductive filament is nanometer scale and buried in the device^38^.

To further examine the resistive switching characteristics, the dependence of resistivity derived from differential resistance on *P*_Ar_ during deposition is plotted in Fig. 2d. The differential resistivities of the as-deposition state at a voltage of − 1.0 V in Region F, and an HRS and LRS around 0.1 V in Regions 2 and 1 of the first cycle, respectively, are compared. The resistivity of the as-deposited state exhibits a clear trend since the increased *P*_Ar_ produces low resistivity, which is consistent with the *P*_Ar_ dependence of the O/Ga ratio identified by the XPS analysis (Fig. 1f). In contrast, the resistivities at the HRS and LRS are almost constant regardless of the *P*_Ar_, suggesting that oxygen vacancies in the as-deposited state are redistributed by applying voltage, and only the enrichment or depletion of oxygen vacancies close to the Pt electrode dominantly determines the resistivity at the HRS and LRS.

**Figure 3:**
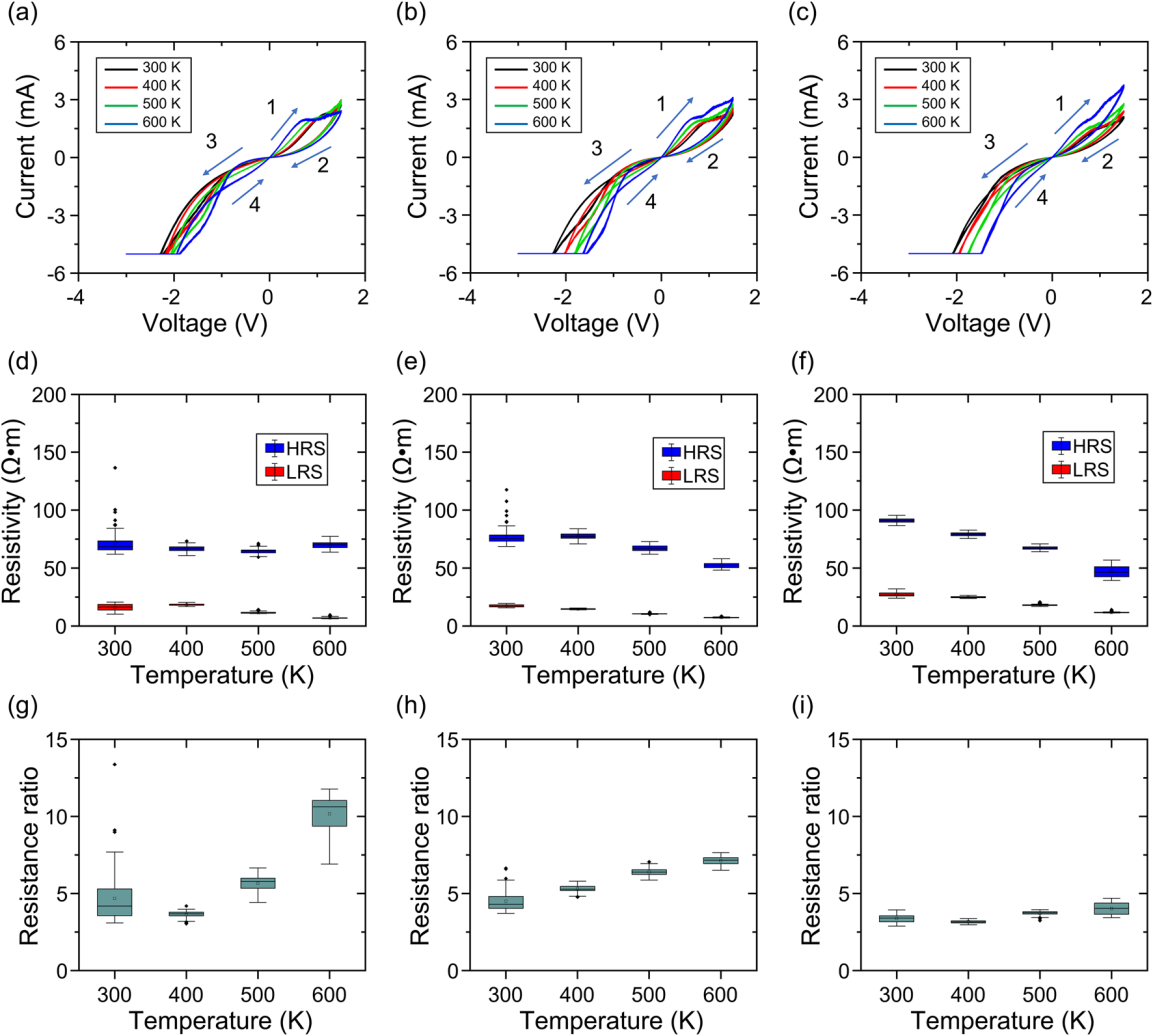
Temperature-dependent properties from 300 to 600 K. *I–V* curves of samples (**a**) G05, (**b**) G10, and (**c**) G15; HRS and LRS resistivity of samples (**d**) G05, (**e**) G10, and (**f**) G15; resistance ratio of samples (**g**) G05, (**h**) G10, and (**i**) G15.

Schematics of the expected oxygen vacancy distribution in the device for the states in Regions 1–4 are shown in Fig. 2(e-1)–(e-4), respectively. When a positive (negative) voltage is applied to the Pt electrode, positively charged oxygen vacancies are repelled from (drift toward) the Pt electrode, depleting (enriching) the oxygen vacancies at the Pt side of GaO_*x*_, which causes switching to the HRS (LRS). Therefore, in this device, resistive switching mainly occurs on the Pt side. However, a crossing in the *I–V* curve was observed for every sample in the negative voltage region, except for the first loop shown in Fig. 2a–c, indicating an abnormal feature of the hysteresis loop. Generally, a standard CF8 or figure-8 (F8) hysteresis loop is observed when resistive switching occurs only on one side of the electrode interface, whereas an abnormal hysteresis is observed when switching occurs on both sides of the electrode interface^41,42^. The reason for switching on both sides in the present device could be the oxidization of GaO_*x*_ near the GaO_*x*_/ITO interface. Since it is theoretically proven that Ga–O has higher bond energy than In–O and Sn–O^43–45^, GaO_*x*_ in contact with the ITO electrode is likely to capture oxygen from ITO, resulting in the depletion of oxygen vacancies in GaO_*x*_ near the ITO interface. Hence, we infer that in Regions 1 and 2 of the positive voltage application (Fig. 2(e-1),(e-2)), the oxygen-vacancy-depleted layer is screened by oxygen vacancies accumulated on the ITO side, whereas the layer is unveiled as the oxygen vacancies drift to the Pt side in the transition of Regions 3–4 (Fig. 2(e-3) to (e-4)). In such a case, resistive switching occurs at both the Pt and ITO sides, and the resistance is switched from the LRS to the HRS when a negative voltage is applied, making a crossing in the hysteresis loop. Such abnormal characteristics can be improved by changing the conditions of bias voltage application, compliance current setting, and ITO composition.

### Temperature dependence of electrical property

The *I–V* characteristics at 300, 400, 500, and 600 K for samples G05, G10, and G15 are shown in Fig. 3a–c, respectively, where the last 10 of 100 loops are plotted. Resistive switching phenomena were observed even at the highest temperature of 600 K for all samples, proving that the oxygen vacancies still function as dopants in a high-temperature environment. The voltage value reaching a compliance current of − 5 mA became smaller as the temperature increased. This suggests that thermal energy activates the ionic conduction of oxygen vacancies, overcoming the energy barrier required for the SET operation.

The temperature dependence of resistivity for the HRS and LRS are summarized in Fig. 3d–f. The differential resistivity was calculated at 0.1 V in Regions 2 and 1 of the *I–V* curve. The results show a decrease in resistivity for both the HRS and LRS as temperature increases. Refer to Supplementary Fig. S5 of Supplementary Information for the Arrhenius plot analysis of the temperature-dependent conductivity. This semiconductor-like feature is consistent with the temperature dependence of non-filamentary-type gallium oxide memristors^19^ and inconsistent with filament-type memristors that show increased resistivity as temperature increases^46^. Therefore, the above results indicate that these devices are driven by not metallic-filament-like but bulk semiconductor-like conduction.

The resistance ratios (RR) obtained from the HRS and LRS resistivities of each sample are shown in Fig. 3g–i. RRs of 3–5 were obtained at 300 K. Conversely, at 600 K, the RR values were approximately 10 for Sample G05 and 4 for Sample G15. The difference in RR among the samples is strongly correlated to the HRS resistivity at high temperatures, and the almost unchanged HRS resistivity of G05 produced a high RR, whereas the significantly decreased HRS resistivity of G15 produced a low RR. Therefore, the results suggest that a moderate density of oxygen vacancies is essential for achieving a high RR at high temperatures.

**Figure 4:**
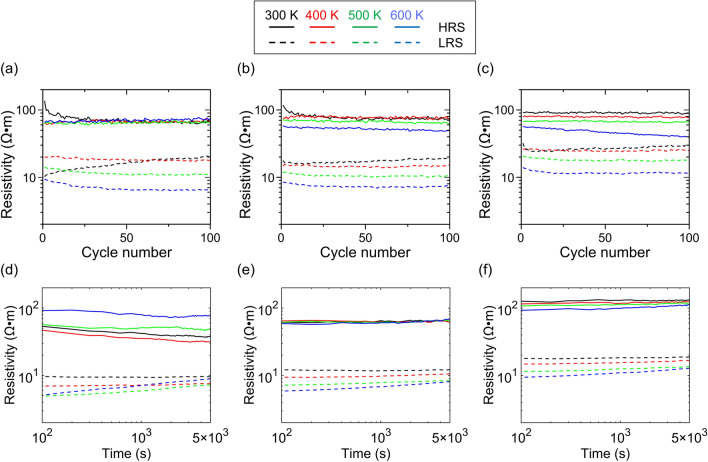
Temperature-dependent properties from 300 to 600 K. *I–V* sweep repeatability of samples (**a**) G05, (**b**) G10, and (**c**) G15, and retention properties of samples (**d**) G05, (**e**) G10, and (**f**) G15.

The *I–V* sweep repeatability and retention properties were also evaluated to examine the device’s reliability. In the repeatability test, voltage sweeps were applied in the order of 0 →  − 3 → 0 →  + 1.5 → 0 V per cycle, and the resistivities at the HRS and LRS were obtained from the measured differential resistances at 0.1 V for Regions 2 and 1, respectively. For the retention test, the device was SET (RESET) to the LRS (HRS) by sweeping the voltage in the order of 0 →  − 3 → 0 (0 →  + 1.5 → 0) V, and the resistivity was obtained from the resistance measured by periodically applying a read voltage of 0.1 V. The results for repeatability and retention are respectively shown in Fig. 4a–c,d–f. Neither characteristic exhibited particular differences among the three types of samples, and there was no particular tendency for the characteristics to deteriorate owing to the high temperature. The resistivity was maintained even after the 100-cycle *I–V* sweeps, and the LRS and HRS were stably maintained at every temperature, which confirmed that the operation of these devices with high reproducibility was demonstrated even at the high temperature of 600 K.

### Conduction mechanism

**Figure 5:**
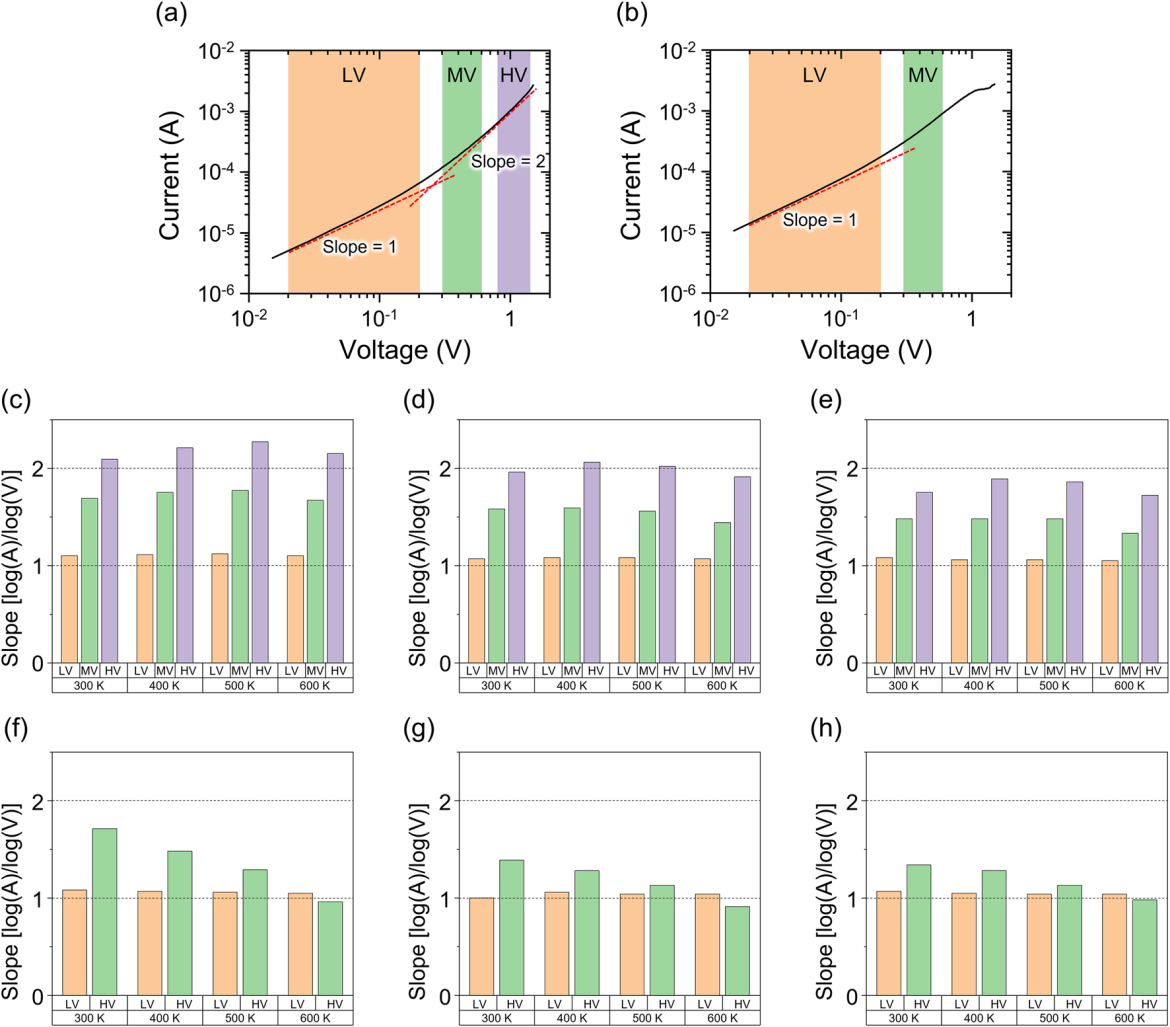
Double logarithmic *I–V* curves of sample G05 for (**a**) HRS and (**b**) LRS with eye-guiding lines for the slope = 1, 2 [log(A)/log(V)]. Values of the slope at HRS and LRS of Samples (**c**,**f**) G05, (**d**,**g**) G10, and (**e**,**h**) G15 at temperatures of 300–600 K.

To elucidate the conduction mechanism between 300 and 600 K, we evaluated the slope of the double logarithmic *I–V* curves. Figure 5a,b show the double-logarithmic *I–V* curves of regions 2 (for HRS) and 1 (for LRS) of sample G05 at 300 K, respectively. For deriving a slope, the *I–V* curves were classified into three regions: low-voltage (0.02 ~ 0.2 V: LV), medium-voltage (0.3 ~ 0.6 V: MV), and high-voltage (0.8 ~ 1.2 V: HV). Note that only the slopes in the LV and MV were derived for the LRS because a negative resistance occurred in the HV. Regarding the slopes, we deduce the conduction mechanism of either the current based on Ohm’s law with a slope of 1 or space-charge-limited current (SCLC) based on the Mott–Gurney law^47^ with a slope of 2. Figure 5c–e,f–h summarize the values of the slope at the HRS and LRS, respectively, of the three samples for all voltage ranges and temperatures. In the LV (orange bars), the slopes were approximately unity for every case, indicating that the ohmic current was dominant irrespective of the resistance state (HRS or LRS), sample type, or temperature. In HV at HRS (violet bars), the slope is close to 2 for samples G05 and G10, indicating the transition from ohmic current to SCLC as the applied voltage increased. The SCLC evolves under a high carrier injection state, particularly at the high-voltage applications in Regions 1 and 2. Such highly injected electrons fill the defect levels near the electrode interface, resulting in the generation of SCLC. In the case of sample G05, the defect levels caused by the oxygen vacancies may act as trap sites that are fully filled with the injected electrons, causing distinct resistance switching between ohmic current and SCLC conduction. The detailed validation of the conduction mechanism is described in Supplementary Information (refer to Supplementary Fig. S5).

In contrast, the slope value of Sample G15 in HV was less than 2, which could be related to G15 having a greater oxygen vacancy density than G05 and G10 since the abundant defect levels caused by the oxygen vacancies are not sufficiently filled with injected electrons, thereby causing imperfect switching between the ohmic current and SCLC and a relatively low RR, as shown in Fig. 3i. Thus, a moderate oxygen vacancy density is necessary for superior high-temperature performance with a high RR and distinct ohmic current/SCLC switching. The slope at MV (green bars) for each device shows values between those of the LV and HV. Hence, ohmic current/SCLC switching occurs through MV as the defect level gradually becomes filled or vacant.

Finally, we discuss the slopes in the MV (green bars) at the LRS, as shown in Fig. 5f–h. In every sample, these were observed to vary with temperature, unlike those at the HRS. This is because SCLC becomes less dominant while the ohmic current contribution increases as the temperature rises, probably due to electrons being de-trapped from the defect levels.

These results indicate that the transition between ohmic current and SCLC works well at 600 K. In addition, we revealed that excess oxygen vacancies deteriorate the ohmic current/SCLC switching and RR. Therefore, optimizing the oxygen vacancy density is essential for achieving high-temperature performance in GaO_*x*_ memristors.

## Conclusion

In this study, Pt/GaO_*x*_/ITO memristors were fabricated, and high-temperature operation was demonstrated. The *I–V* hysteresis loops of the non-filamentary-type switching, supported by the semiconductor-type temperature dependence of resistivity, were confirmed at high temperatures of up to 600 K, which, to our knowledge, is the record-high operating temperature for GaO_*x*_ memristors. The O/Ga composition ratio *x* in GaO_*x*_ sensitively influenced the resistivity and resultant resistance ratio of the memristor. Meanwhile, the *I–V* sweep repeatability and retention property were less dependent on *x* and exhibited highly stable behavior between 300 and 600 K. We found that the distinct ohmic current and SCLC conduction mechanisms function even at 600 K, demonstrating the highly temperature-durable resistive switching characteristics of the GaO_*x*_ memristors. These results reveal that GaO_*x*_ holds promises for enabling the high-temperature operation of neuromorphic computing systems.

## Methods

GaO_*x*_ films were deposited using a PLD instrument equipped with a sintered Ga_2_O_3_ target and light source using a fourth-harmonic Nd:YAG laser (*λ* = 266 nm). The RHEED patterns were observed in situ to confirm the amorphous phase of GaO_*x*_. Circular Pt electrodes with diameters of 50 μm were deposited by sputtering using a metal mask. The GaO_*x*_ thicknesses of G05, G10, and G15 were 100, 80, and 60 nm, respectively. XPS analysis was performed to investigate the effect of Ar pressure on the O/Ga ratio and the Ga valence state. The temperature dependence of the electrical properties was measured using a semiconductor parameter analyzer (Keysight Technologies B1500A) and a stage temperature controller (LakeShore Model 335). The *I–V* measurements were performed in a vacuum chamber by applying voltages to the Pt electrode, while the ITO electrode was grounded by changing the sample stage temperature from 300 to 600 K at 100 K intervals. A maximum temperature of 600 K was chosen as the temperature at which the amorphous phase was sufficiently maintained^48,49^.

## Supplementary Information

Supplementary Information.

## Data Availability

The data supporting this study’s findings are available in the article and its supplementary material.
