# Supplementary material for: High-temperature operation of gallium oxide memristors up to 600 K
Source: Sci Rep. 2023 Jan 30;13:1261. doi: 10.1038/s41598-023-28075-4 (PMC9886979; doi:10.1038/s41598-023-28075-4)
Supplement: Supplementary file 1 — Supplementary Information. [file 41598_2023_28075_MOESM1_ESM.pdf]

Supplementary Information for
High-temperature operation of gallium oxide memristors up to 600 K

Kento Sato, Yusuke Hayashi*, Naoki Masaoka, Tetsuya Tohei and Akira Sakai*

Graduate School of Engineering Science, Osaka University, 1-3 Machikaneyama-cho, Toyonaka, Osaka, 560-8531, Japan

*Corresponding author (Y.H.): Phone: +81-6-6850-6302; Email: hayashi@ee.es.osaka-u.ac.jp
*Corresponding author (A.S.): Phone: +81-6-6850-6300; Email: sakai@ee.es.osaka-u.ac.jp

The details of *I*–*V* characteristics.
To confirm the endurance characteristics of 100-cycle *I*–*V* sweep, the resistive switching behavior of Samples G05, G10, and G15 was characterized at room temperature with a sweep rate of 1.1 V/s and current compliance of −5 mA for 100 cycles in total, as shown in Fig. S1. The first *I*–*V* curve at a negative voltage sweep (0→ −3 → 0 V) was performed, and then the subsequent *I*–*V* sweep (0 → +1.5 → 0 → −3 → 0 V) was repeated for 100 cycles, as shown in Fig S1. Note that Figs. 2(a)–2(c) are the first 10 cycles of Fig. S1(a)–(c). Consequently, the samples exhibited good endurance through the 100-cycle *I*–*V* sweep.

We investigate the *I*–*V* characteristics starting from the negative and positive voltage applications, as shown in Fig. S2. Compared with the regular *I*–*V* sweep starting from the negative voltage (0 → −3 → 0 V) [Fig. S2(a)], the first *I*–*V* sweep starting from the positive voltage (0 → +1.5 → 0 V) showed little current flow and no hysteresis characteristics [Fig. S2(b)]. The first positive-voltage application produced several-microamperes output current with Schottky-junction characteristics [Fig. S2(c)]. These results indicate that negative voltage application is required for resistive switching. This phenomenon could be related to the high turn-on voltage required for the first SET operation. Once the first negative voltage is applied on the device, the electrical barrier at the Pt/GaO_x interface is eliminated by the reduction of GaO_x surface via oxygen vacancy supplied from the ITO electrode side, resulting in the evolution of three-order higher current flow and hysteresis *I*–*V* characteristics.

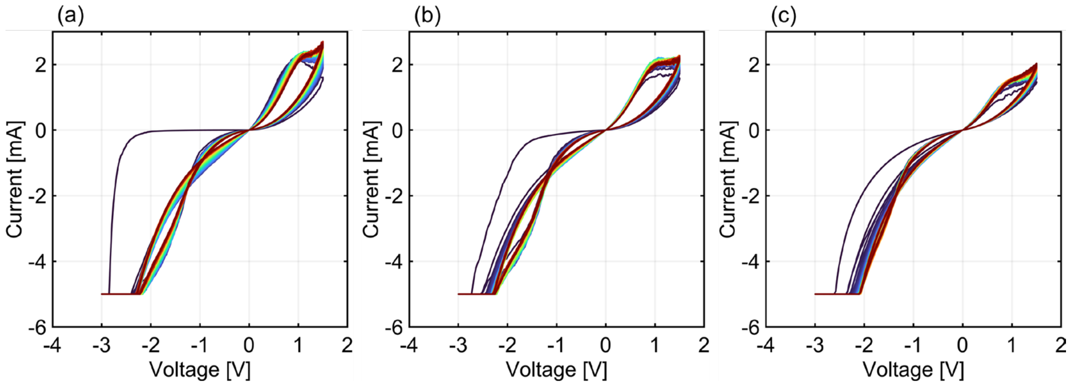

Fig. S1. 100-cycle *I*–*V* curves of Samples (a) G05, (b) G10, and (c) G15.

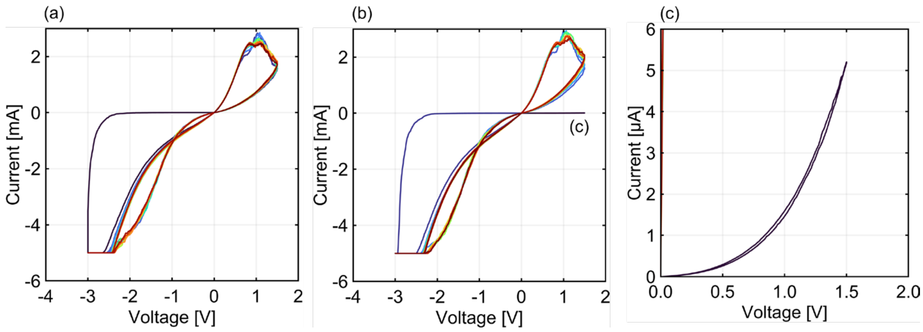

Fig. S2 *I*–*V* characteristics of Sample G05 starting from the (a) negative and (b) positive voltage sweeps. (c) The latter produced several-microamperes output current with Schottky-junction characteristics in the first positive-voltage application.

Electrode area dependency.

To verify bulk conduction type resistive switching of gallium oxide-based memristors, Aoki *et al*. and Kura *et al*. reported a linear relationship between the output current and electrode area [1, 2]. Contrary to those reports, the present study did not exhibit clear electrode area dependence of output current, as shown in Fig. S3 for Sample G10 with electrode diameters of 150 and 50 μm. The possible cause is due to the in-plane inhomogeneity of oxygen vacancy distribution. We infer that a non-uniform current path was formed via the high turn-on voltage. Such high turn-on voltage is considered to induce the area-dependent leakage current, e.g., at the edge of the circular electrode, resulting in the in-plane inhomogeneous segregation of oxygen vacancy via resistive switching. Given this assumption, spatially non-uniform distribution of Pt/GaO_x Schottky barrier height potentially weakens the area dependency of current flow.

To support the non-filamentary conduction mechanism in the present study, we refer to our recent work reported by Masaoka *et al*.[3]; a scaling down of output current I against electrode area S was demonstrated via the fabrication of a crossbar array a-GaO_x memristors as shown in Fig. S4 (these devices are referred to as Sample GX), where the slopes of double logarithmic plot in I-S characteristic are 0.63 for LRS and 0.65 for HRS. In Masaoka's work, we deposited the a-GaO_x films using the same PLD system as the present study and similar deposition conditions with those for Samples G05, G10, and G15. Consequently, the improved scaling-down feature is attributable to the voltage application protocol, where a gradual increase in sweep range could help to avoid the high turn-on voltage and enhance the in-plane homogeneous current flow. A comprehensive investigation remains for future work.

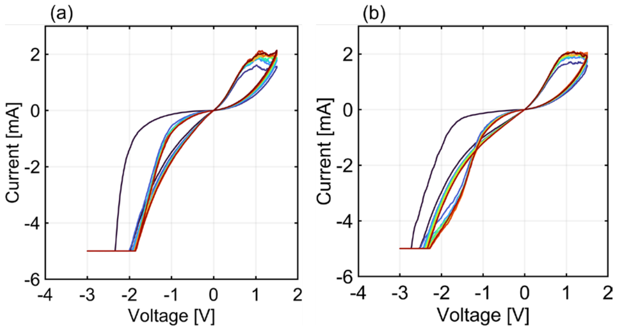

Fig. S3. 10-cycle I–V curves of G10 with a Pt electrode diameters of (a) 150 and (b) 50 μm.

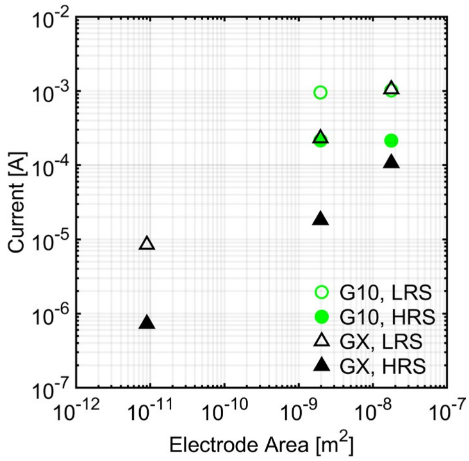

Fig. S4 Electrode area dependence of output current values of Samples G10 and GX.

Arrhenius plot of conductivity.

Based on the temperature-dependent resistivity of Samples G05, G10, and G15 [Figs. 3(d)–3(f)], the Arrhenius plot of conductivity σ, the inverse of resistivity, is depicted in Fig. S5. From the slope of the Arrhenius plot from 300 K to 600 K, the activation energy E_a of ohmic conduction was extracted for samples G05, G10, and G15: $E_a = 40$, 42, and 40 meV for the LRS and 2, 18, and 31 meV for the HRS. These values compare favorably with 78 meV in a previous study on GaO_x memristors[4], indicating that a similar defect level due to oxygen vacancies was formed in the present study.

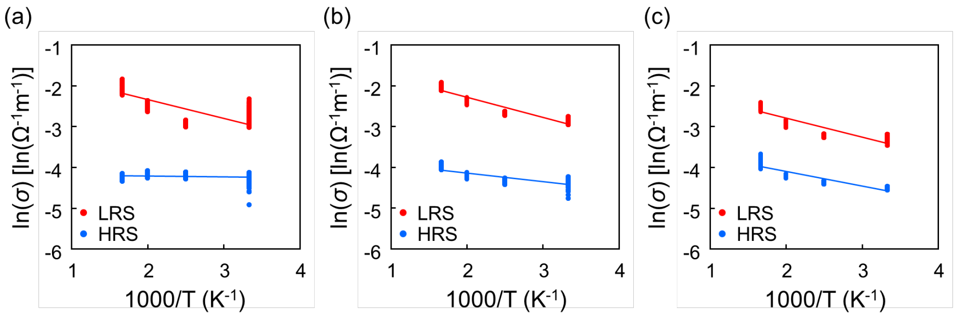

Fig. S5. Arrhenius plot of conductivity for Samples (a) G05, (b) G10, and (c) G15.

Detailed validation of the conduction mechanism

We performed a detailed analysis of the *I*–*V* curve based on the SCLC formula[5]. The carrier density n_0 and electron mobility μ_n were extracted by fitting with the following formulae.

$$J_{\mathrm{Ohm}} = q n_0 \mu_n \frac{V}{d_s}, \quad (1)$$

$$J_{\mathrm{SCL}} = \frac{9}{8}\epsilon_0 \epsilon_{\mathrm{r}} \mu_n \frac{V^2}{d_s^3}, \quad (2)$$

where q is the elementary charge, d_s is the film thickness where SCLC occurs (in this case, the entire film thickness), ε_0 is the permittivity of vacuum, and ε_r is the relative permittivity of Ga_2O_3. We conducted the curve fitting at LV and HV regions of HRS to obtain the carrier density and electron mobility. Consequently, n_0 = [1.62×10^{16}, 1.90×10^{16}, and 2.28×10^{16} cm^{-3}] and μ_n = [5.66×10^{-2}, 5.31×10^{-2}, and 4.82×10^{-2} cm^{2}V^{-1}s^{-1}] were derived at 300 K for Samples [G05, G10, and G15] (Fig. S6). Compared to the values $n_0 = 2\times10^{14}$ cm^{-3} and μ_n = 8 cm^{2}V^{-1}s^{-1} reported in a previous study of a-GaO_x [6], the carrier density is two orders of magnitude larger, and the electron mobility is two orders of magnitude smaller. Meanwhile, the resistivity values of these results are consistent: 68 Ωm for Sample G05 and 39 Ωm for the previous study. Therefore, the values obtained in this fitting are considered reasonable.

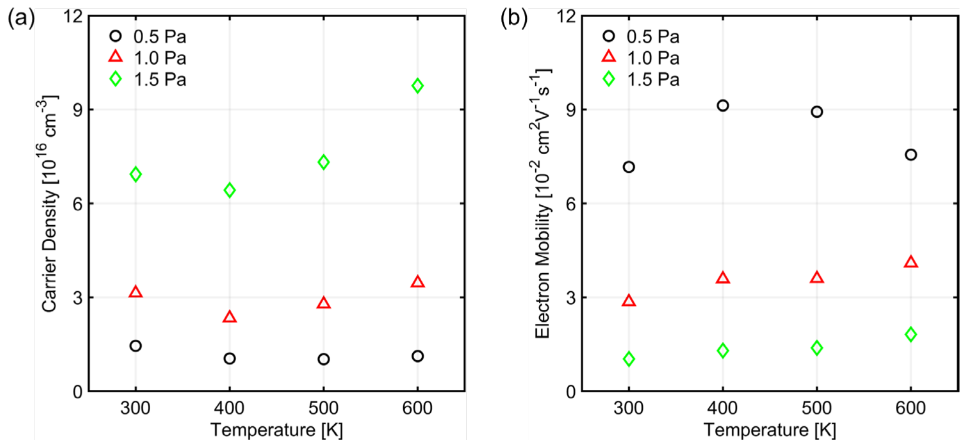

Fig. S6 carrier density and electron mobility of Samples G05, G10, and G15 obtained via fitting to *I*–*V* curves.

References

[1]. Kura, C., Aoki, Y., Tsuji, E., Habazaki, H., & Martin, M., Fabrication of A Resistive Switching Gallium Oxide Thin Film with A Tailored Gallium Valence State and Oxygen Deficiency by RF Cosputtering Process *RSC Adv.* **6**, 8964 (2016).

[2]. Aoki, Y., Wiemann, C., Feyer, V., Kim, H.-S., Schneider, C. M., Ill-Yoo, H., & Martin, M., Bulk Mixed Ion Electron Conduction in Amorphous Gallium Oxide Causes Memristive Behaviour *Nat. Commun.* **5**, 1 (2014).

[3]. Masaoka, N., Hayashi, Y., Tohei, T., & Sakai, A., Interface Engineering of Amorphous Gallium Oxide Crossbar Array Memristors for Neuromorphic Computing *Jpn. J. Appl. Phys.* **62**, (2023). DOI:10.35848/1347-4065/acb060.

[4]. Gao, X., Xia, Y., Ji, J., Xu, H., Su, Y., Li, H., Yang, C., Guo, H., Yin, J., & Liu, Z., Effect of Top Electrode Materials on Bipolar Resistive Switching Behavior of Gallium Oxide Films *Appl. Phys. Lett.* **97**, 193501 (2010).

[5]. Kim, K. M., Choi, B. J., Lee, M. H., Kim, G. H., Song, S. J., Seok, J. Y., Yoon, J. H., Han, S., & Hwang, C. S., A Detailed Understanding of The Electronic Bipolar Resistance Switching Behavior in Pt/TiO_2/Pt Structure *Nanotechnology* **22**, 254010 (2011).

[6]. Kim, J., Sekiya, T., Miyokawa, N., Watanabe, N., Kimoto, K., Ide, K., Toda, Y., Ueda, S., Ohashi, N., Hiramatsu, H., Hosono, H., & Kamiya, T., Conversion of An Ultra-Wide Bandgap Amorphous Oxide Insulator to A Semiconductor *NPG Asia Mater.* **9**, e359 (2017).
